# Antibacterial and Antibiofilm Photodynamic Activities of Lysozyme-Au Nanoclusters/Rose Bengal Conjugates

**DOI:** 10.1021/acsomega.1c00838

**Published:** 2021-03-24

**Authors:** Ichie Okamoto, Hirofumi Miyaji, Saori Miyata, Kanako Shitomi, Tsutomu Sugaya, Natsumi Ushijima, Tsukasa Akasaka, Satoshi Enya, Satoshi Saita, Hideya Kawasaki

**Affiliations:** † Department of Periodontology and Endodontology, Faculty of Dental Medicine, Hokkaido University, N13 W7, Kita-ku, Sapporo, Hokkaido 060-8586, Japan; ‡ Division of Periodontology and Endodontology, School of Dentistry, Health Sciences University of Hokkaido, 1757 Kanazawa, Tobetsu-cho, Ishikari-gun, Hokkaido 061-0293, Japan; § Support Section for Education and Research, Faculty of Dental Medicine, Hokkaido University, N13 W7, Kita-ku, Sapporo, Hokkaido 060-8586, Japan; ∥ Department of Biomedical Materials and Engineering, Faculty of Dental Medicine, Hokkaido University, N13 W7, Kita-ku, Sapporo, Hokkaido 060-8586, Japan; ⊥ Department of Chemistry and Materials Engineering, Faculty of Chemistry, Materials and Bioengineering, Kansai University, 3-3-35 Yamate-cho, Suita-shi, Osaka 564-8689, Japan

## Abstract

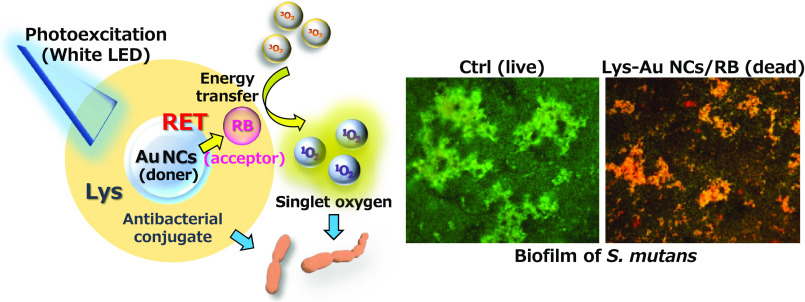

Antibacterial photodynamic therapy (aPDT) utilizes reactive oxygen species such as singlet oxygen (^1^O_2_) and free radicals via photosensitizers, which are light and light-sensitive agents, to reduce bacterial infections. It has been utilized as a treatment for dental diseases in place of antibiotic therapies. However, aPDT does not always cause the desired therapeutic effect due to the instability of organic photosensitizers and the formation of bacterial biofilms. To promote the antibacterial and antibiofilm effects of aPDT, we have proposed a lysozyme (Lys)-gold nanoclusters (Au NCs)/rose bengal (Lys-Au NCs/RB) conjugate as a novel photosensitizer. This conjugate was found to effectively impede the growth of both gram-positive and gram-negative bacteria when exposed to white light-emitting diode (LED) irradiation. The photoexcited Lys-Au NCs/RB showed significantly higher antibacterial activity than photoexcited Lys-Au NCs or RB alone. The synergistic effect is a result of the combination of Lys (an antibacterial protein) and enhanced ^1^O_2_ generation related to resonance energy transfer (RET) in the Au NCs/RB conjugate. Photoexcited Lys-Au NCs/RB increased the effects of aPDT in a dose- and time-dependent manner. Furthermore, the photoexcited Lys-Au NCs/RB successfully decreased *Streptococcus mutans* biofilm formation. However, in contrast, it did not have a negative effect on the proliferation, adhesion, or spread of mammalian cells, indicating low cytotoxicity. Lys-Au NCs/RB is a novel photosensitizer with low cytotoxicity that is capable of bacterial inactivation and the suppression of biofilm formation, and could help to improve dental treatments in the future.

## Introduction

Antibacterial photodynamic therapy (aPDT) is widely used alongside oral disinfection therapies, including those for caries, endodontic disease, periodontitis, and peri-implantitis. The use of conventional antibiotics has the potential to promote bacterial drug resistance.^1^ In addition, bacterial biofilms, which typically form on the tooth surface, frequently impede drug penetration into the biofilm.^2^ With aPDT, a photosensitizer generates singlet oxygen (^1^O_2_) from ground state dioxygen, which exerts antibacterial effects on gram-positive and gram-negative bacterial cells.^3−6^ In contrast to antibiotic therapy, aPDT rarely produces drug-resistant bacteria and may contribute to the destruction of the biofilm matrix.^7−11^ Thus, aPDT has the potential to serve as an effective antibacterial treatment to solve conventional antibiotic therapy problems in dentistry.

Organic dyes such as methylene blue (MB) have been employed as substrates for photoexcitation in aPDT.^12^ Organic photosensitizers show high antibacterial activity; however, such photosensitizers require a narrow spectrum of excitation wavelengths to generate ^1^O_2_.^12^ Recently, various antibacterial nanocomposites have been developed to replace the conventional procedures.^13−17^ Kawasaki et al. previously reported that Au_25_(SR)_18_ nanoclusters (H – SR = captopril) (Au NCs) generated ^1^O_2_ via photodynamic effects.^18^ Compared to organic dyes, Au NCs have the physical advantage of employing a much wider spectrum of absorption wavelengths (400–900 nm) for photoexcitation.^18^ Furthermore, Miyata et al. revealed that Au NCs exhibit good cytocompatibility and high photostability, as well as antibacterial effects, when compared to conventional organic dyes.^19^ Hence, novel photosensitizers that incorporate Au NCs are expected to be useful in aPDT.^20^

Resonance energy transfer (RET) is a widely known phenomenon in which energy is transferred between two light-sensitive molecules.^21^ The complex of Au NCs and an organic dye should be able to absorb visible wavelength light, such as that produced by low-cost white light-emitting diode (LED) devices, to effectively promote the generation of ^1^O_2_ via the RET mechanism. Yamamoto et al., to investigate this, created a photosensitizer composed of Au NCs and MB and obtained evidence for the occurrence of RET in the resulting conjugate.^22^ The authors suggested that the light energy absorbed by the Au NCs was transferred to MB to generate large amounts of ^1^O_2_. However, MB is a potential pollutant; notably, the accumulation of MB in water bodies has been shown to have adverse health effects.^23^ Numerous studies have already reported the use of Au NC-based photosensitizers for aPDT,^20^ but the effects of Au NC-based photosensitizers on the growth of biofilms remains unknown.

**Scheme 1:**
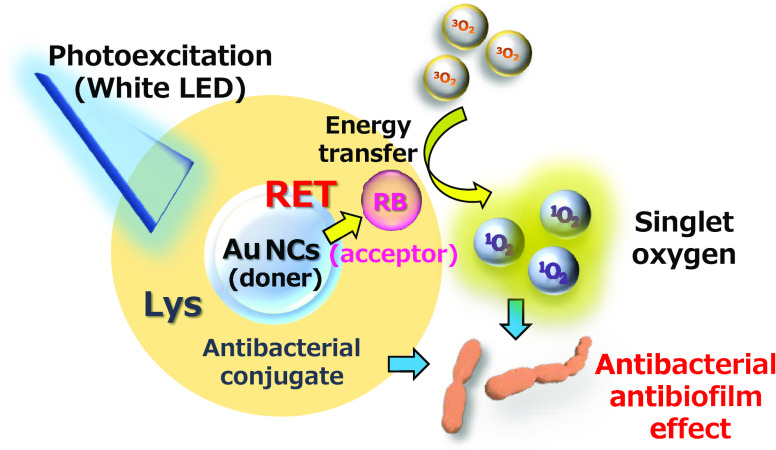
Schematic Illustration of Photodynamic Inactivation Mechanisms of a Photoexcited Lys-Au NCs/RB Conjugate

For these reasons, the current study sought to create novel Au NCs photosensitizers that were protected by lysozyme (Lys) and used rose bengal (RB) as a photosensitive dye. Compared to RB or Au NCs alone, the resulting conjugate, designated lysozyme-Au nanoclusters/rose bengal (Lys-Au NCs/RB), demonstrated excellent aPDT action and destruction of the oral bacteria’s biofilms. The development process for the Lys-Au NCs/RB is shown in Scheme 1 and focused on the following aspects: (i) RB is widely used as an aPDT photosensitizer in dentistry because of its low toxicity, excellent water solubility, and high ^1^O_2_ generation efficiency (nearly 75%); the RB can bind to specific sites of Lys,^24^ producing the Lys-Au NCs/RB. (ii) Lys is a widely known biological protein with antibacterial properties^25^ and was recently shown to decrease biofilms.^26^ Thus, compared to RB alone, a composite of the RB photosensitizer with Lys was expected to provide increased aPDT action while decreasing biofilms; and (iii) an RET process from Au NCs to RB is expected in Lys-Au NCs/RB, as the emission spectra of the donor (Au NCs) and absorption spectra of the acceptor (RB) overlap. This RET process is expected to enhance the aPDT activity of RB.^22^ In the present study, we evaluated whether Lys-Au NCs/RB with white LED irradiation exerted antibacterial and antibiofilm activities against oral bacteria, and in particular, when supported by the RET mechanism. In addition, we assessed the cytotoxicity of Lys-Au NCs/RB against mammalian cells to elucidate the biosafety properties for the potential application of the conjugate in a clinical setting.

## Results and Discussion

### Characterization of Lys-Au NCs/RB

**Figure 1:**
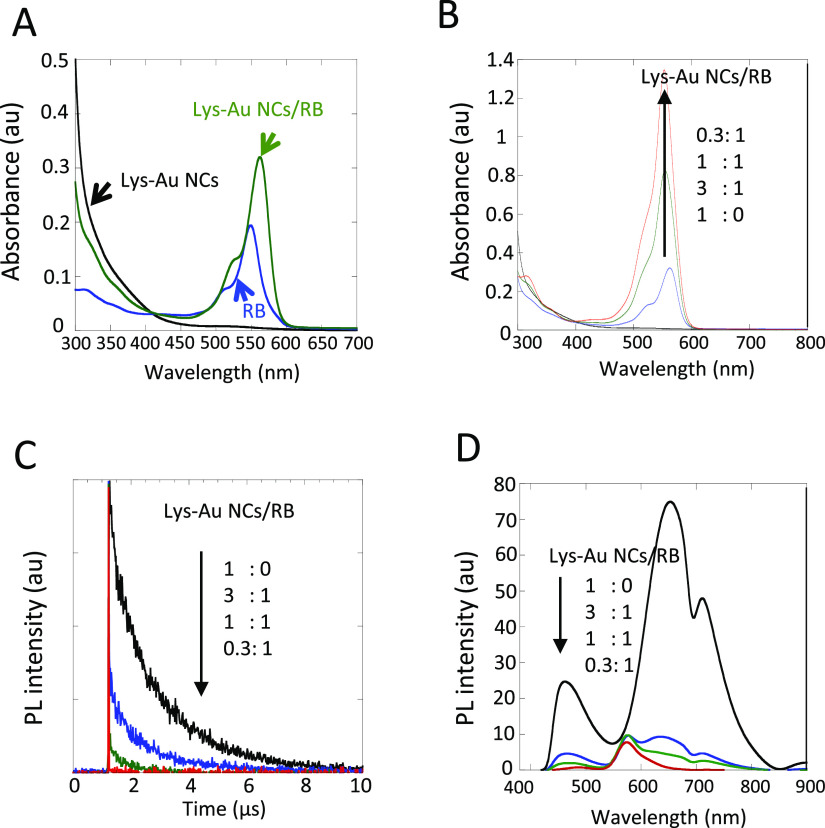
Characterization of the Lys-Au NCs/RB conjugate. (A) UV–vis spectra of Lys-Au NCs and the Lys-Au NCs/RB conjugate. (B) UV–vis spectra of Lys-Au NCs and the Lys-Au NCs/RB conjugate at various ratios of Lys-Au NCs to RB. (C) Fluorescence decay curves for Lys-Au NCs and the Lys-Au NCs/RB conjugate at various ratios of Lys-Au NCs to RB, as assessed using excitation/emission wavelengths of 365/650 nm. (D) Fluorescence emission spectra of Lys-Au NCs and the Lys-Au NC/RB conjugate at various ratios of Lys-Au NCs, as assessed using excitation/emission wavelengths of 365/650 nm. Abbreviations: au, arbitrary unit; Lys-Au NCs, lysozyme-Au nanoclusters; PL, photoluminescence; RB, rose bengal; UV–vis, ultraviolet–visible.

The absorption and fluorescence spectra of Lys-Au NCs, RB, and the Lys-Au NCs/RB conjugate are shown in Figure 1A. No apparent surface plasmon resonance absorption peak at 520 nm was observed for the Lys-Au NCs, suggesting the formation of small Au NCs of less than 2 nm in size. It is well-known that Au nanoparticles more than 3 nm in size will exhibit a surface plasmon resonance absorption peak. When excited by light at 370 nm, the suspension of Lys-Au NCs showed an emission peak centered at 650 nm. This observation was consistent with the known optical properties of the Lys-Au NCs.^27^ Upon conjugation of RB with Lys-Au NCs, the RB absorption was observed at approximately 550 nm, in addition to absorption at less than 450 nm by Lys-Au NCs; the absorbance of RB in the conjugates increased with the ratio of RB to Lys-Au NCs (Figure 1B). This effect indicates the conjugation of Lys-Au NCs and RB via the interaction of Lys and RB.^28^ From the DLS measurements, the size (*D*) of the Lys-Au NCs/RB conjugate (Lys-Au NCs: RB = 0.3:1) was estimated to be 12 nm, which is larger than that of Lys alone (D ≈ 4 nm). This increase presumably reflects the formation of Lys-Au NCs/RB by conjugation.

### RET from Lys-Au NCs to RB

In the Lys-Au NCs/RB, we expected RET from Lys-Au NCs to RB due to the spectra overlapping between the donor (Lys-Au NCs) emission and the acceptor (RB) absorption.^29^ The adsorption band of RB at 550 nm overlaps with the emission spectrum of Lys-Au NCs from 430 to 580 nm, satisfying the overlap condition needed for RET. Fluorescence lifetime measurements have often been used to confirm the occurrence of RET as it decreases the average luminescence lifetime of the donor, concomitant with a decrease in the fluorescence intensity of the donor. Thus, we conducted fluorescence time-resolved experiments on Lys-Au NCs/RB, and the analysis based on curve-fitting methods is shown in Figure 1C. The fluorescence decay of the Lys-Au NCs (the donor) at 650 nm at the excitation wavelength of 365 nm was monitored (Table 1). The fluorescence lifetimes of the Lys-Au NCs/RB decreased with the increases in the RB to Au NCs ratio. The average decay time (∼1.87 μs) of Lys-Au NCs decreased to 0.29 μs for the Lys-Au NCs/RB conjugate (0.3:1), together with a decrease in the fluorescence intensity of the Au NCs (Figure 1D). These observations suggest that there is RET from the Au NCs to the RB in the Lys-Au NCs/RB. Such energy transfer via RET should improve the efficiency of ^1^O_2_ generation by Lys-Au NCs/RB and their PDT actions. The calculated RET efficiencies are also shown in Table 1, assuming that the change in the average decay time of the Lys-Au NCs/RB conjugate originates from the RET process and the use of decay time of Au NCs in the presence and absence of RB.^22^ The RET efficiencies from the Au NCs to the RB depend on the content of RB in Lys-Au NCs/RB conjugates; as the ratio of RB in Lys-Au NCs/RB conjugates increases, the RET efficiency also increases.

**Table 1:** Parameters for Lys-Au NCs and the Lys-Au NCs/RB Conjugate^a^

entry	Em	<*t*>	*t*_1_(*A*_1_)	*t*_2_ (*A*_2_)	Φ_ET_ (%)
Lys-Au NCs	650 nm	1.87	0.475(0.37)	2.06(0.63)	
(Au:RB) 3:1	650 nm	1.38	0.009(0.88)	1.44(0.12)	26
(Au:RB) 1:1	650 nm	0.66	0.052(0.95)	0.75(0.05)	65
(Au:RB) 0.3:1	650 nm	0.29	0.003(0.99)	0.48(0.01)	85

a *t*_1_ and *t*_2_ are the fluorescence lifetimes (μs), and *A*_1_ + *A*_2_ = 1. ⟨*t*⟩ is the average fluorescence lifetime (μs). Abbreviation: Lys-Au NCs, lysozyme-Au nanoclusters; RB, rose bengal. Φ_ET_ (%): RET efficiencies from the Au NCs to the RB in Lys-Au NCs/RB conjugates.

### ^1^O_2_ Generation by Lys-Au NCs/RB Following Illumination with a White LED

**Figure 2:**
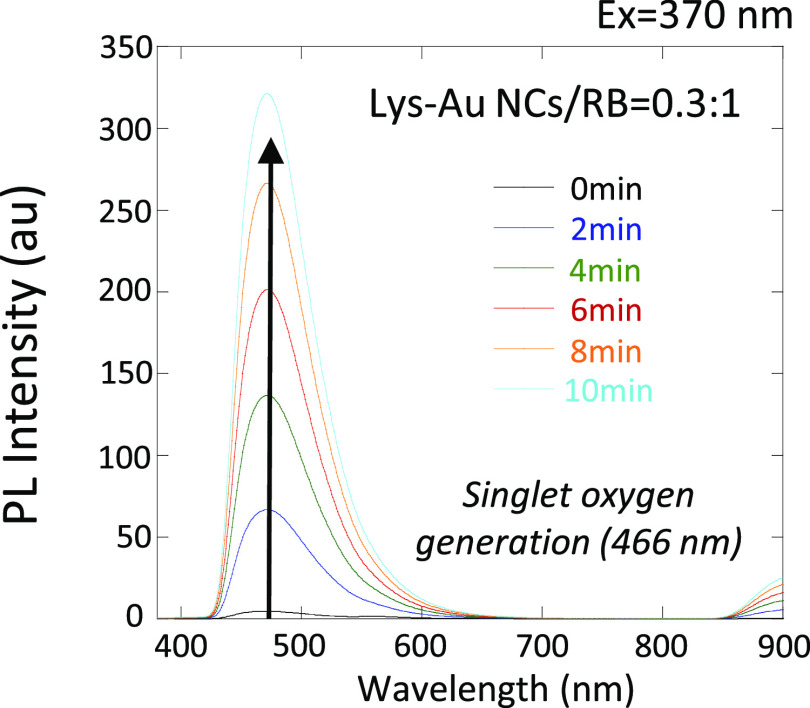
Detection of ^1^O_2_. Fluorescence spectra of the MTX at an excitation wavelength of 370 nm in the presence of the Lys-Au NCs/RB conjugate (0.3:1) under white LED irradiation. Abbreviations: au, arbitrary unit; LED, light-emitting diode; Lys-Au NCs, lysozyme-Au nanoclusters; MTX, methotrexate; PL, photoluminescence; RB, rose bengal.

We evaluated ^1^O_2_ generation by photoexcited Lys-Au NCs/RB in the D_2_O with MTX. A white LED light was used as the light source because of the visible absorbance of the Lys-Au NCs/RB. The photoexcited Lys-Au NCs/RB produced ^1^O_2_, resulting in the oxidation of MTX. As a result, an increase in the fluorescence intensity at 466 nm was observed over time under light irradiation (Figure 2). The ^1^O_2_ quantum yields (Φ_NC/RB_) of Lys-Au NCs/RB were estimated to be 0.20, 0.47, and 0.59 using RB (^1^O_2_ quantum yield Φ_RB_ = 0.75) as a standard for Lys-Au NCs:RB ratios of 3:1, 1:1, and 0.3:1, respectively. Thus, at higher RB ratios, the value of the ^1^O_2_ quantum yield increased. It is likely that RET from Au NCs to RB enhanced ^1^O_2_ generation by RB in the Lys-Au NCs.

### aPDT and RET Effects of the Lys-Au NCs/RB Conjugate

**Figure 3:**
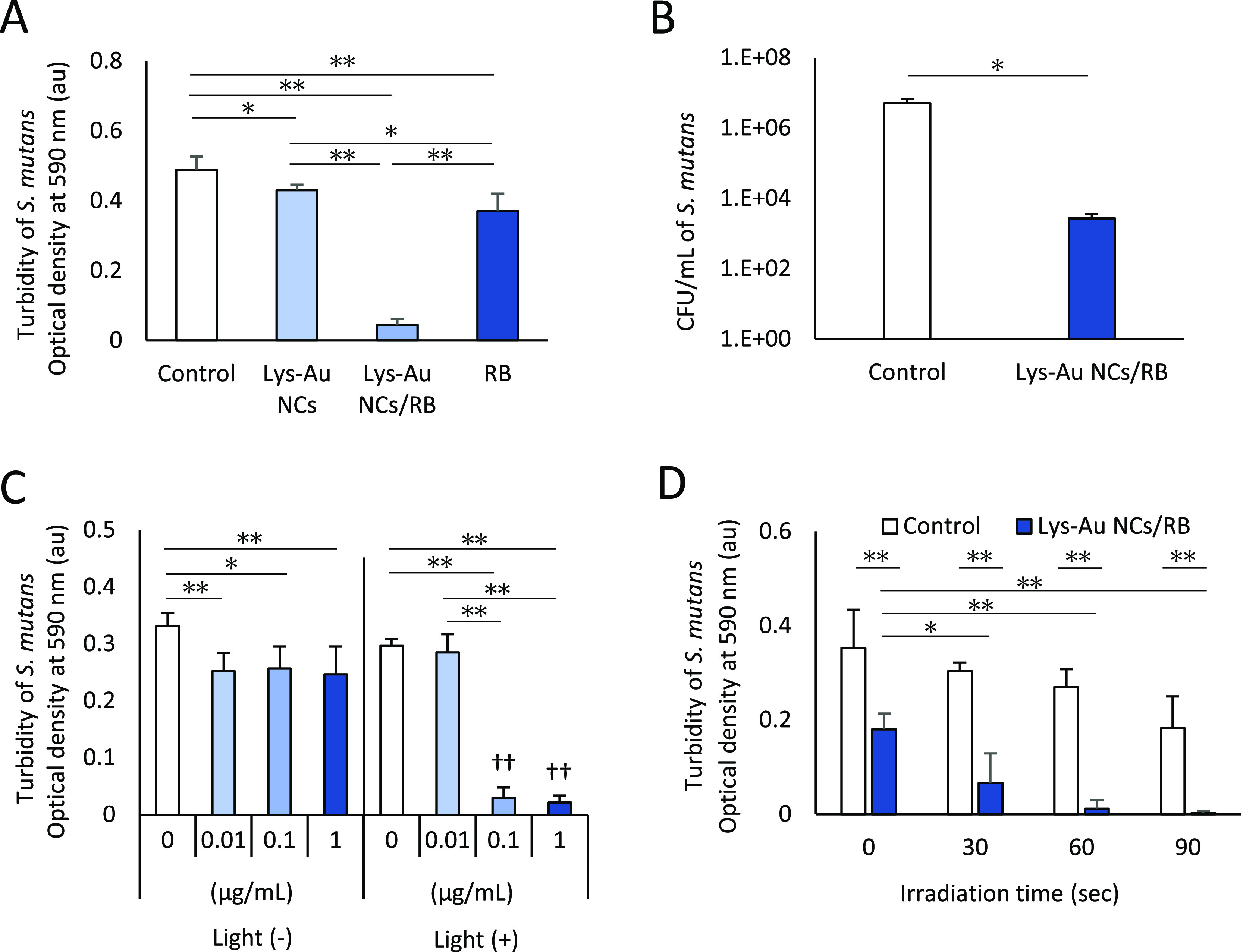
Antibacterial characterization of the Lys-Au NCs/RB conjugate. (A) Turbidities of *Streptococcus mutans* cultured without treatment (control) or exposed to Lys-Au NCs, Lys-Au NCs/RB, or RB (*n* = 6). Statistical analysis: one-way ANOVA with Tukey HSD post hoc test (*, *P* < 0.05; **, *P* < 0.01). (B) Cell counts (CFU/mL) of *S. mutans* cultured without treatment (control) or exposed to Lys-Au NCs/RB (*n* = 3). Statistical analysis: Student’s *t* test (*, *P* < 0.05). (C) Turbidities of *S. mutans* cultures exposed to increasing doses of Lys-Au NCs/RB with or without LED irradiation (*n* = 6). Statistical analysis: One-way ANOVA with Tukey HSD post hoc test (*, *P* < 0.05; **, *P* < 0.01). (D) Turbidities of *S. mutans* cultures irradiated at increasing intervals without (control) or with Lys-Au NCs/RB (*n* = 6). Statistical analysis: one-way ANOVA with Tukey HSD post hoc test (*, *P* < 0.05; **, *P* < 0.01). Values are presented as mean ± standard deviation. Abbreviations: au, arbitrary unit; CFU, colony-forming unit; LED, light-emitting diode; Lys-Au NCs, lysozyme-Au nanoclusters; RB, rose bengal.

To assess the antibacterial effects of the photoexcited Lys-Au NCs/RB (Lys-Au NCs:RB = 0.3: 1), the turbidities of the *S. mutans* suspensions were measured to compare the viabilities of the cultures exposed to photoexcited Lys-Au NCs, Lys-Au NCs/RB, or RB (Figure 3A). The turbidities of the bacterial suspensions grown in the absence of supplementation (control) or in the presence of Lys-Au NCs, Lys-Au NCs/RB, or RB were 0.49, 0.43, 0.05, and 0.37, respectively. Significantly lower turbidities were obtained for bacterial suspensions grown in the presence of photosensitizers when compared to the control (Lys-Au NCs vs control: *P* < 0.05, Lys-Au NCs/RB, or RB vs control: *P* < 0.01). In addition, we confirmed that turbidity in the presence of Lys-Au NCs/RB was significantly lower than that in the presence of Lys-Au NCs or RB alone (*P* < 0.01). Assessments of CFUs of *S. mutans* showed that exposure to photoexcited Lys-Au NCs/RB significantly (*P* < 0.05) decreased the CFU count of *S. mutans* by 1000-fold when compared to the cultures lacking Lys-Au NCs/RB (Figure 3B). Based on these results, we inferred that Lys-Au NCs/RB photoexcited by white LED exerted antibacterial activity against *S. mutans*. We hypothesize that photoexcited Au NCs and RB generate ^1^O_2_, leading to bactericidal effects. Reactive oxygen species (ROS) such as ^1^O_2_, namely, oxidative stress, are known to cause damage to bacterial cell DNA and membranes,^30,31^ leading to bacterial cell death. ROS stimulate events related to cell death, including apoptosis, autophagy, and ferroptosis, through an increase in lipid peroxidation.^32^

In addition, Lys-Au NCs/RB increased antibacterial activity compared to RB alone, suggesting that RET between Au NCs and RB is relevant in aPDT. White LED irradiation is known to emit a wide range of wavelengths, extending from 400 to 900 nm and therefore is expected to excite both Au NCs and RB. Given the evidence (Figure 1 and Table 1) that photoexcited Au NCs emit energy that is transferred to RB, ^1^O_2_ production by RB is likely to be increased following photoexcitation, thereby increasing the antibacterial action. The RET mechanism may potentiate the antibacterial effects, and thus may contribute to the future development of aPDT applications. A previous study proposed the enhancement of biological aPDT effects via RET.^33^ Yuan et al. showed that the bioluminescence emitted by luminol was absorbed by another photosensitizer (oligo(*p*-phenylene vinylene)) to produce ROS via RET, resulting in the killing of both bacterial and cancer cells.^34^ Such a RET system is expected to expand the utility of aPDT. The limitation of RB as a photosensitizer has a negative impact on cell uptake due to the hydrophilic character of RB, which makes it difficult for this dye to cross bacterial membranes during cellular uptake.^35^ However, the interaction of Lys with *S. mutans* has been demonstrated previously.^36^ Such an interaction is also expected for Lys-Au NCs/RB and *S. mutans*, which may enhance aPDT action on *S. mutans.* Investigation of the interactions between Lys-Au NCs/RB and *S. mutans* would be informative but are beyond the scope of this investigation.

### Dose- and Time-Dependent Effects of aPDT Using the Lys-Au NCs/RB Conjugate

To assess the dose-dependent effects of aPDT when using Lys-Au NCs/RB, the suspension of *S. mutans* was combined with Lys-Au NCs/RB at a range of concentrations (0 (absence), 0.01, 0.1, and 1 μg/mL), both in the presence and absence of LED irradiation. In the case of no irradiation, exposure to Lys-Au NCs/RB resulted in a mild but statistically significant (*P* < 0.05) decrease in the turbidity of *S. mutans* cultures, regardless of the Lys-Au NCs/RB concentration. With LED irradiation, exposure to Lys-Au NCs/RB at concentrations of 0, 0.01, 0.1, and 1 μg/mL resulted in turbidities of 0.29, 0.28, 0.03, and 0.02, respectively. Thus, photoexcited 0.1 and 1 μg/mL Lys-Au NCs/RB significantly reduced the turbidity of *S. mutans* cultures (*P* < 0.01, Figure 3C)*.* These results suggested that Lys-Au NCs/RB at concentrations of 0.1 μg/mL or greater was effective for aPDT in this experimental system. Previously, Miyata et al. evaluated the antibacterial activity of Au NCs (Au_25_(Capt)_18_) irradiated with a blue LED,^19^ and they found that the antibacterial effects against *S. mutans* required Au NCs at a concentration of 500 μg/mL. Moreover, Yamamoto et al. obtained improved antibacterial activity with Au NCs encapsulated in BSA-capped Au NCs conjugated with MB (BSA-Au NC-MB conjugate);^22^ antibacterial effects against *S. mutans* required 100 μg/mL of a BSA-Au NC-MB conjugate when irradiated by a white LED. Thus, our Lys-Au NCs/RB conjugate appears to possess greater antibacterial potency against *S. mutans* than that obtained with Au NCs alone or with other reported Au NC-based conjugates. We speculate that, in addition to the organic dye RB, the antibacterial Lys protein also contributes to the antibacterial activity of Lys-Au NCs/RB. This inference is consistent with the results of Wu et al., who reported that the addition of Lys improved the antibacterial effects of chitosan nanoparticles.^37^

To assess the time-dependent effects of LED irradiation, *S. mutans* suspensions supplemented with the Lys-Au NCs/RB conjugate (at 0 (absence) or 1 μg/mL) were subjected to white LED irradiation for 0, 30, 60, and 90 s. The results showed that each irradiation time significantly reduced the turbidity of *S. mutans* compared to that of the control (absence of Lys-Au NCs/RB; *P* < 0.01)*.* These data are consistent with the above observation that incubation with the conjugate, even in the absence of irradiation, resulted in decreased turbidity. In addition, irradiation for any non-zero interval (30, 60, and 90 s) significantly diminished the turbidity compared to a no-irradiation sample (30 s vs 0 s: *P* < 0.05; 60 or 90 s vs 0 s: *P* < 0.01, Figure 3D). Hence, it appears that Lys-Au NCs/RB provides irradiation-time-dependent enhancement of the antibacterial effects against *S. mutans*. These results were consistent with those of the MTX-based analysis, which demonstrated that longer irradiation resulted in an increased generation of ^1^O_2_ (Figure 2).

### Effects of the Photoexcited Lys-Au NCs/RB Conjugate on Other Bacterial Species

**Figure 4:**
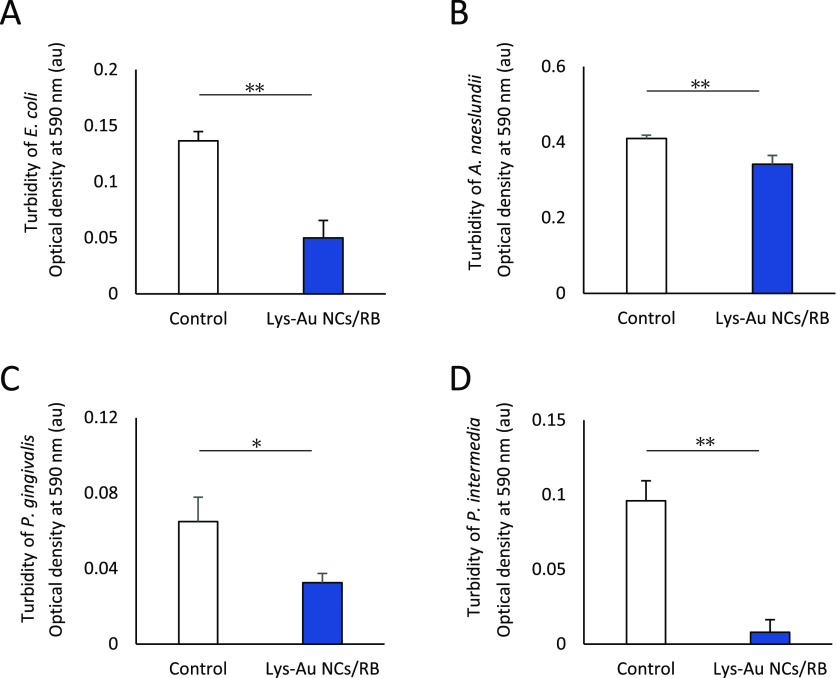
Antibacterial activity of the Lys-Au NCs/RB conjugate against several species of bacteria. Turbidities (mean ± standard deviation) of *Escherichia coli* (A), *Actinomyces naeslundii* (B), *Porphyromonas gingivalis* (C), and *Prevotella intermedia* (D) cultured without (control) and with Lys-Au NCs/RB (*n* = 6). Statistical analysis: Student’s *t* test (*, *P* < 0.05; **, *P* < 0.01). Abbreviations: au, arbitrary unit; Lys-Au NCs, lysozyme-Au nanoclusters; RB, rose bengal.

The photoexcited Lys-Au NCs/RB conjugate consistently decreased the turbidity of cultures for several other bacterial species, including *E. coli* (*P* < 0.01), *A. naeslundii* (*P* < 0.01), *P. gingivalis* (*P* < 0.05), and *P. intermedia* (*P* < 0.01), compared with the control cultures (lacking Lys-Au NCs/RB) (Figure 4A-D). Hence, it appears that the photoexcited Lys-Au NCs/RB conjugate possesses antibacterial activity against both gram-negative and gram-positive cells. *A. naeslundii* is known as the primary colonizer of the tooth surface.^38^ Notably, inhibition of the growth of this species would decrease dental biofilm formation and potentially prevent dental disease. The other two tested species, *P. gingivalis* and *P. intermedia*, are obligate anaerobic bacteria that have been isolated from the oral cavities of patients with periodontitis or infected root canals.^39,40^ Both of these species must be cultured under anaerobic conditions, meaning that these bacteria grow in a medium containing low residual concentrations of oxygen. Nonetheless, the use of Lys-Au NCs/RB with subsequent irradiation resulted in decreased turbidities for cultures of both *P. gingivalis* and *P. intermedia*. Lys has been reported to inhibit the coaggregation of *P. gingivalis*(41) and the activities of lipopolysaccharides produced by *P. gingivalis* and *P. intermedia*.^42^ Hence, the antibacterial activity of the Lys-Au NCs/RB conjugate under low-oxygen conditions may have involved these additional effects of the Lys component, in addition to ^1^O_2_ generation. However, further research is needed to elucidate the relationship between Lys-Au NCs/RB activity and residual oxygen.

### Morphological Observations

**Figure 5:**
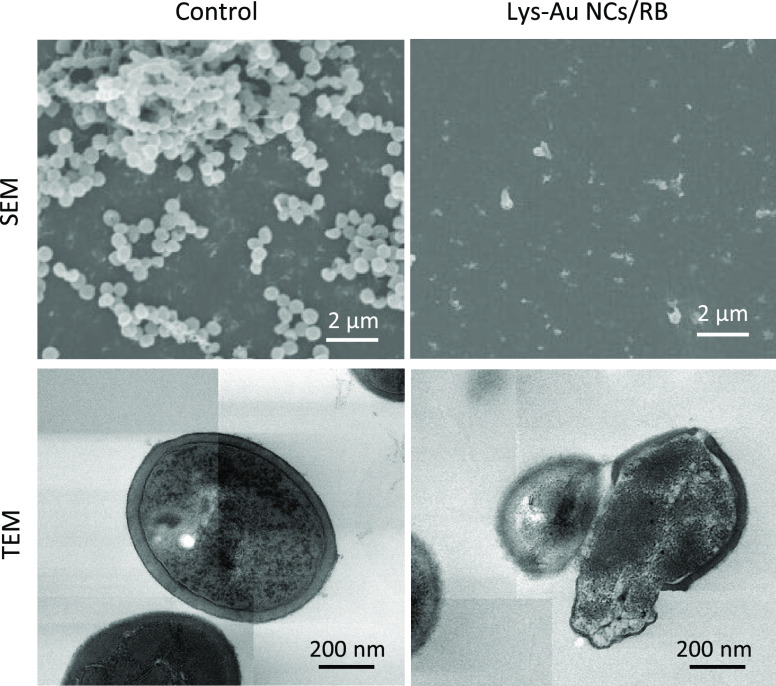
Morphological examinations. Representative SEM and TEM images of *Streptococcus mutans* cultured without (control) and with Lys-Au NCs/RB. Scale bars represent 2 μm in the SEM images and 200 nm in the TEM images. Abbreviations: SEM, scanning electron microscope; TEM, transmission electron microscope; Lys-Au NCs, lysozyme-Au nanoclusters; RB, rose bengal.

For morphological assessments, SEM and TEM observations of the *S. mutans* exposed to the Lys-Au NCs/RB conjugate and subjected to LED irradiation were made (Figure 5). SEM images of the control cultures (lacking Lys-Au NCs/RB) revealed the presence of chains of normally shaped *S. mutans* cells. However, after exposure to Lys-Au NCs/RB and irradiation, deformed bacterial cells were frequently observed. In the TEM images, control *S. mutans* cultures (lacking Lys-Au NCs/RB) showed spherical cell bodies enclosed by intact cell membranes. In contrast, cultures grown following the addition of Lys-Au NCs/RB and irradiation exhibited bacterial cell bodies that were frequently irregular and possessed damaged cell membrane structures. It appears that ^1^O_2_ generated by photoexcited Lys-Au NCs/RB attacks the cell membrane, resulting in decreased cell growth.

### Effects of Photoexcited Lys-Au NCs/RB Conjugate on Bacterial Biofilms

**Figure 6:**
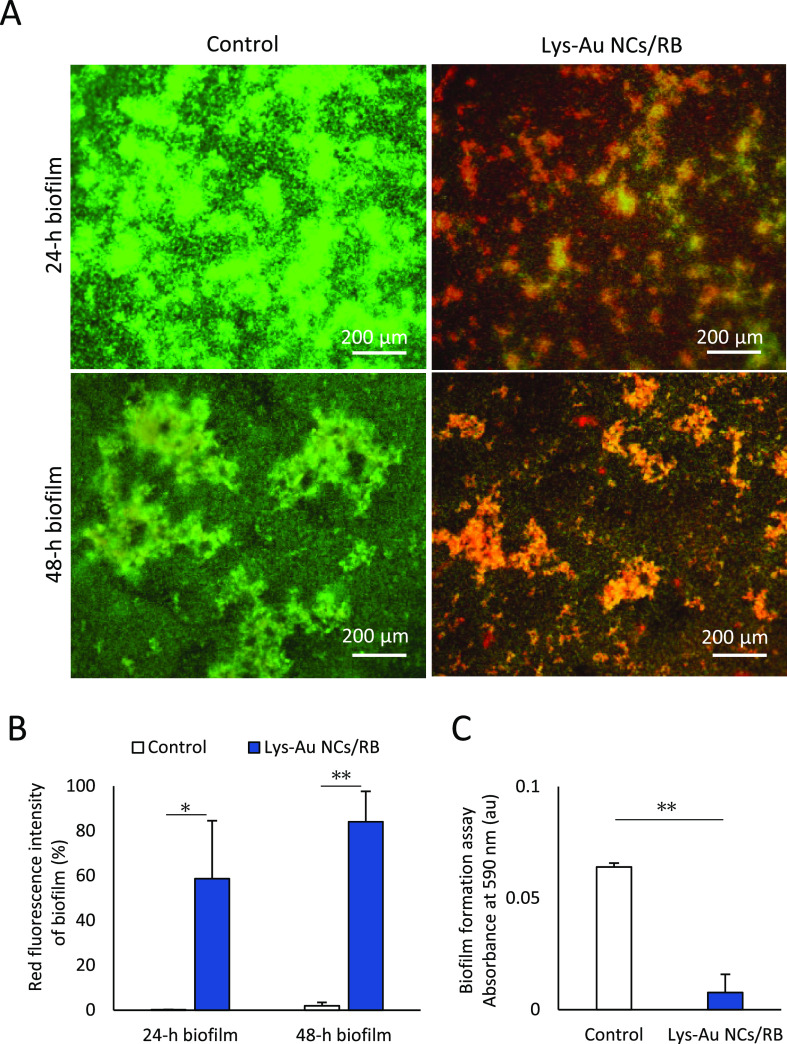
Biofilm inhibition assessments. (A) LIVE/DEAD BacLight staining of biofilms of *Streptococcus mutans* cultured for 24 and 48 h in the absence (control) or presence of Lys-Au NCs/RB. (B) Red fluorescence intensity of LIVE/DEAD BacLight staining for the 24 and 48 h*-*incubated biofilms of *S. mutans* cultured in the absence (control) or presence of Lys-Au NCs/RB. (mean ± standard deviation, *n* = 3). Statistical analysis: Student’s *t* test (*, *P* < 0.05; **, *P* < 0.01). (C) Formation assay of *S. mutans* biofilm cultured in the absence (control) or presence of Lys-Au NCs/RB. Values are presented as mean ± standard deviation (*n* = 6). Statistical analysis: Student’s *t* test (**, *P* < 0.01). Scale bar represents 200 μm. Abbreviations: au, arbitrary unit; Lys-Au NCs, lysozyme-Au nanoclusters; RB, rose bengal*.*

When *S. mutans* biofilms were cultured for 24 or 48 h after aPDT using Lys-Au NCs/RB, LIVE/DEAD BacLight staining frequently revealed the presence of red-staining bacteria, which indicates dead cells. In contrast, the control cultures rarely showed red-staining of *S. mutans* (Figure 6A). Quantification of the intensity of LIVE/DEAD staining demonstrated significant increases in the degree of red staining at 24 h (*P* < 0.05) and 48 h (*P* < 0.01), when compared to that of the controls (Figure 6B). In addition, a biofilm formation assay showed that exposure to the photoexcited Lys-Au NCs/RB conjugate significantly attenuated the increase in biofilm volume (*P* < 0.01) (Figure 6C). These results suggest that photoexcited Lys-Au NCs/RB effectively destroyed the bacterial biofilms. Destruction of bacterial biofilms is a key strategy for attacking inflammatory dental diseases, given the compromised efficacy of antibiotics in treating biofilms.^43^ Previous reports have shown that aPDT destroys cells in bacterial biofilms.^44^ For instance, Pereira et al. showed that the addition of MB followed by illumination with a low-power laser significantly decreased the viability of bacterial cells in *in vitro* biofilms.^45^ While Darabpour et al. also showed that an Au NCs/MB conjugate exhibited significant anti-biofilm photoinactivation against *S. aureus.*(46) These authors speculated that the MB dye was able to penetrate deeper layers of the biofilm. We hypothesize that appropriate combinations of antibacterial and photosensitizing materials will be important to fully realize aPDT’s ability to destroy biofilms, in place of the antibiotics. Further research is needed regarding the nanotechnology and drug delivery system.

### Cytotoxicity Evaluation

**Figure 7:**
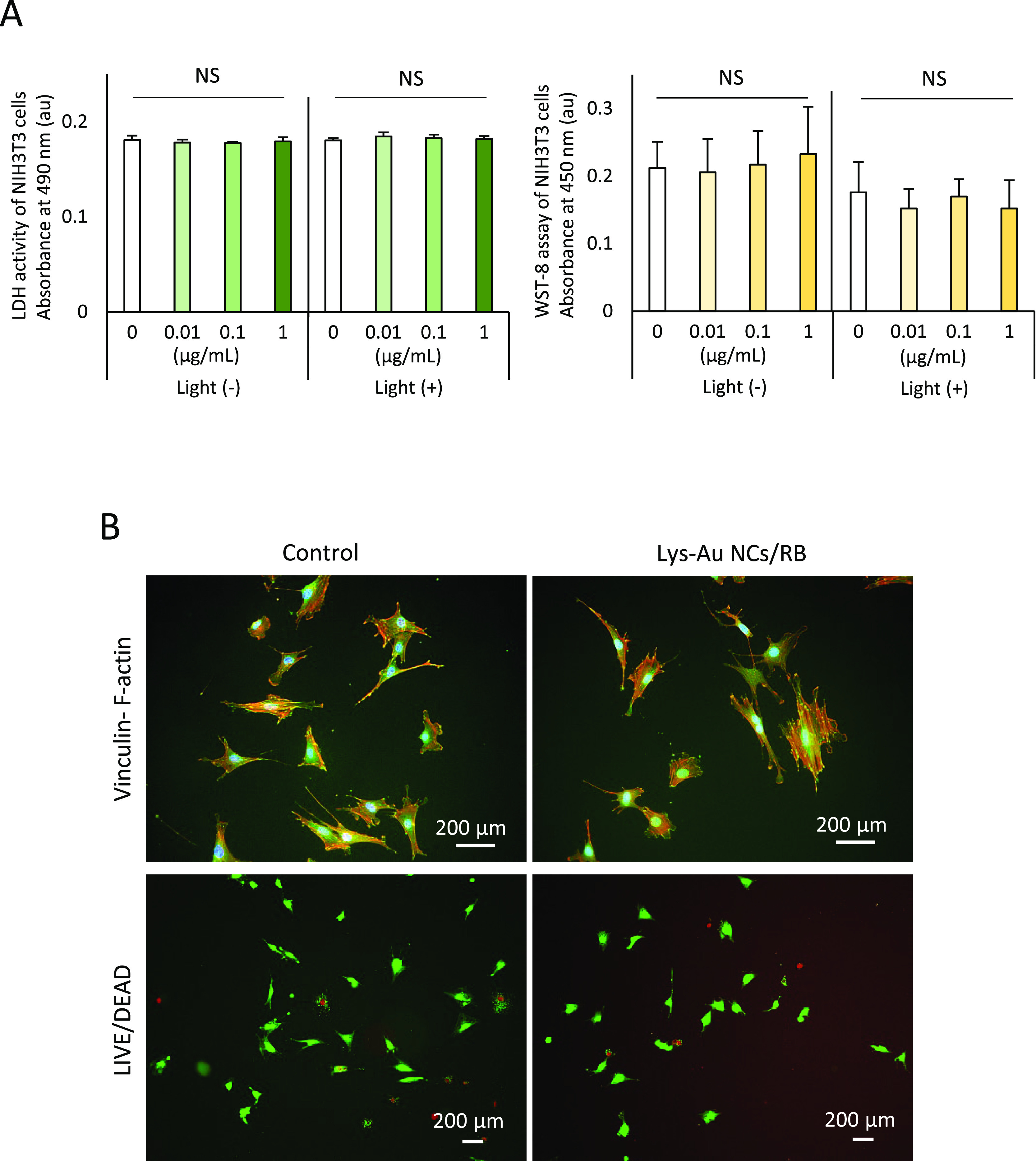
Cytotoxicity assessments. (A) WST-8 and LDH activities of NIH3T3 cells cultured with increasing doses of Lys-Au NCs/RB with or without LED irradiation (mean ± standard deviation, *n* = 6). Statistical analysis: one-way ANOVA. (B) Vinculin/F-actin staining and LIVE/DEAD BacLight staining of NIH3T3 cells cultured without (control) or with Lys-Au NCs/RB. Scale bar represents 200 μm. Abbreviations: NS, not significant (*P* ≥ 0.05); LDH, lactate dehydrogenase; Lys-Au NCs, lysozyme-Au nanoclusters; RB, rose bengal; WST-8, water soluble tetrazolium salts-8.

Biomaterials with strong antibacterial activity also exhibit strong cytotoxicity. It is well-known that cytotoxic biomaterials reduce WST-8 and increase LDH activity. In the present study, WST-8 and LDH assays of mammalian cells showed no significant differences (*P* ≥ 0.05) between the control (no application) and Lys-Au NCs/RB conjugate-exposed cultures of NIH3T3 cells (Figure 7A). Fluorescence observations for the vinculin-F-actin double staining, which is associated with cell adhesion, revealed similar cell shapes in both the control and aPDT-exposed cultures and normal spreading on the culture dishes (Figure 7B upper). In the LIVE/DEAD staining, both the control and aPDT cultures showed cells emitting green fluorescence (live cells) (Figure 7B lower). Hence, it seems likely that Lys-Au NCs/RB has low cytotoxicity. Previously, Miyata et al. compared the biocompatibility properties of Au NCs and a conventional organic dye, MB, at clinically relevant antibacterial concentrations.^19^ This study showed that exposure to MB decreased the viability of fibroblastic and osteoblastic cells; exposure to Au NCs did not. It is thus important that the cytotoxicity of organic dyes in aPDT be reduced. In a similar previous investigation, conjugation to photosensitizers was shown to decrease the required amount of organic dye, improving cytocompatibility. Specifically, Shitomi et al. reported that Ag NCs complexed with RB exhibited good cytocompatibility while retaining antibacterial activity.^47^ The release of antibacterial Ag^+^ ions from Ag NCs decreased the required amount of RB, thereby decreasing cytotoxicity. We propose that the biosafety properties of the Lys-Au NCs/RB conjugate may be advantageous for dento-medical applications.

## Conclusions

This work investigated the *in vitro* antibacterial and antibiofilm activities and cytocompatibility of Lys-Au NCs/RB following photoexcitation by white LED irradiation. Fluorescence measurements showed that ^1^O_2_ was produced by photoexcitation of the Lys-Au NCs/RB, which is an effect likely mediated by RET from the Au NCs to the RB in Lys-Au NCs/RB. Photoexcited Lys-Au NCs/RB significantly inhibited the growth of all tested species of oral bacteria, including *S. mutans*, *E. coli*, *A. naeslundii*, *P. gingivalis*, and *P. intermedia.* Photoexcited Lys-Au NCs/RB also damaged the *in vitro*-generated biofilms of *S. mutans.* Furthermore, photoexcited Lys-Au NCs/RB showed low cytotoxicity against NIH3T3 fibroblasts. Therefore, photoexcitation of the Lys-Au NCs/RB conjugate with white LED may be useful for aPDT in dental treatments.

## Materials and Methods

### Materials

Tetrachloroauric (III) acid (HAuCl_4_·3H_2_O, 99.99%), methotrexate (MTX, 98%), dimethylformamide (DMF, 99.5%), Lys (for biochemistry and sourced from egg white), methanol (99.9%), RB, and heavy water (D_2_O, 99.9%) were purchased from FUJIFILM Wako Pure Chemical Corporation (Osaka, Japan). We used these chemicals without further purification. Pure water (resistivity: 18.2 MΩ·cm) was prepared using a Barnstead NANO pure DI water system (Cole-Parmer Instrument Company, Vernon Hills, IL, USA).

### Synthesis of Lys-Au NCs

Lys-Au NCs were synthesized according to a modified version of a previously described method.^48^ Briefly, 5 mL of HAuCl_4_ solution (10 mM) was mixed with 5 mL of Lys solution (50 mg/mL) under vigorous stirring for 3 min. The mixed solution was adjusted to pH ≈ 11 by adding 1 M NaOH solution (1 mL). After reaction at 37 °C for 6 h with stirring (500 rpm), an apparent brown suspension of Lys-Au NCs was obtained. The suspension of Lys-Au NCs was passed through a 0.45 μm filter, and the filtered suspension was purified using a centrifugal ultrafiltration tube with a 3000 Da cut-off (Merck Millipore, MA, USA).

### Synthesis of Lys-Au NCs/RB Conjugates

**Figure 8:**
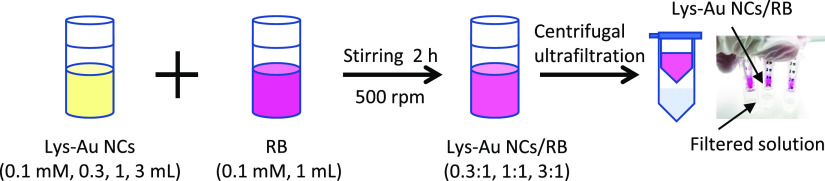
Preparation procedure of Lys-Au NCs/RB.

We prepared Lys-Au NCs/RB conjugates using the interaction of Lys and RB. The preparation procedure of Lys-Au NCs/RB is schematically summarized in Figure 8. Aliquots of 0.1 mM RB (1 mL) were mixed with 3, 1, or 0.3 mL of the 0.1 mM Lys-Au NC suspension, corresponding to the Lys-Au NCs to RB ratios of 3:1, 1:1, or 0.3:1 (respectively). The resulting suspensions were stirred at 500 rpm for 2 h using a magnetic stirrer. The concentration of Lys-Au NCs was defined based on that of the Lys. The suspensions were purified using a centrifugal ultrafiltration tube with a 3000 Da cut-off (Merck Millipore, MA, USA). Note that the filtered suspension did not contain free RB, indicating that it was bound to the Lys-Au NCs.

### Detection of ^1^O_2_

We used a chemical trap ^1^O_2_ probe, MTX for ^1^O_2_ generation from the photoexcited Lys-Au NCs/RB conjugate.^49^^1^O_2_ can oxidize MTX and this produces fluorescent species, which enables the evaluation of ^1^O_2_ generation via the fluorescence increase. Ten millimolars MTX was mixed in DMF with 2 mL of an aqueous (D_2_O) suspension of Lys-Au NCs/RB. The suspensions were then irradiated with a white LED device (15 mW, 80 mW/cm^2^ at 450 nm; Twin-Arm Luminaire SPF-D2; Shodensha, Osaka, Japan) to detect the ^1^O_2_ generated by the conjugate.

The ^1^O_2_ quantum yield of the Lys-Au NCs/RB (Φ_NC/RB_) was calculated using the following equation:
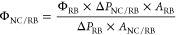
where Φ_RB_ is the ^1^O_2_ quantum yield of RB, which was 0.75; Δ*P*_NC/RB_ and Δ*P*_RB_ are the fluorescence intensity changes of MTX when combined with Lys-Au NCs/RB and RB, respectively; *A*_NC/RB_ and *A*_RB_ represent the light absorbed by the Lys-Au NCs/RB and RB, respectively; and *A*_NC_ and *A*_RB_ were estimated by integrating the absorption bands in the wavelength range of 400–800 nm.

### Absorption and Fluorescence Spectra

Ultraviolet–visible (UV–vis) absorption spectroscopy and steady-state fluorescence spectroscopy measurements were conducted using an ultraviolet–visible–near infrared (UV–vis–NIR) spectrophotometer (Model V-670; JASCO, Tokyo, Japan) and a spectrofluorometer (Model FP-6300; JASCO), respectively. All measurements were performed at room temperature using 1 cm cuvettes. Fluorescence lifetime was measured by time-correlated single-photon counting with a Quantaurus-Tau fluorescence lifetime measurement system (C11367-03; Hamamatsu Photonics Co., Hamamatsu, Japan). Dynamic light scattering (DLS) was performed on a Zetasizer Nano ZS (Malvern Panalytical, Ltd., Malvern, UK) equipped with a He–Ne laser operating at 632.8 nm and a scattering detector at 173°.

### Preparation of Bacterial Suspensions

Strains of bacterial cells, gram-positive facultative anaerobic bacteria (*Streptococcus mutans* ATCC 35668 and *Actinomyces naeslundii* ATCC 27039), gram-negative facultative anaerobic bacteria (*Escherichia coli* ATCC 25922), and gram-negative obligate anaerobic bacteria (*Porphyromonas gingivalis* ATCC 63143627 and *Prevotella intermedia* ATCC 25611) were obtained from the American Type Culture Collection (Manassas, VA, USA). The bacteria were grown anaerobically using an Anaeropack system (Mitsubishi Gas Chemical Company, Inc., Tokyo, Japan) and brain heart infusion broth (Eiken Chemical, Co., Ltd., Tokyo, Japan) supplemented as follows: no supplementation for *E. coli*; 1% sucrose (FUJIFILM Wako Pure Chemical Corporation) and antibiotics; 0.1% gramicidin D and bacitracin (FUJIFILM Wako Pure Chemical Corporation) for *S. mutans*; and 0.5% yeast extract, 0.0005% hemin (Sigma-Aldrich Co. LLC, St. Louis, MO, USA), and 0.0001% menadione (Sigma-Aldrich Co. LLC) for *P. gingivalis* and *P. intermedia*. *A. naeslundii* was grown in Actinomyces broth (Becton Dickinson and Company, Franklin Lakes, NJ, USA). For antibacterial assessments, bacteria in log-phase growth were diluted to final concentrations of approximately 1 × 10^7^ colony-forming units (CFU) per mL.

### Antibacterial Effects of the Lys-Au NCs/RB Conjugate and White LED

For the antibacterial assessments, Lys-Au (0.23 μg/mL), Lys-Au NCs/RB (1 μg/mL), or RB (0.77 μg/mL) was added to a suspensions of *S. mutans*, and the resulting mixture was dispensed into 48-well microplates at 500 μL/well. As the Lys-Au NCs included Au at 25% wt, we used a weight ratio of 1:4.3:3.3 (Lys-Au:Lys-Au NCs/RB:RB) to match the weight ratio of Au NCs and RB. Immediately following distribution into the 48-well plates, the plate was illuminated with white LED irradiation. The LED light was 2 cm from the surface of the bacterial suspension and illuminated it for a period of 1 min. The *S. mutans* samples used as the control received only white LED light (i.e., in the absence of the conjugate and dye). After anaerobic incubation for 24 h, the turbidity of the *S. mutans* suspension was examined using a colorimeter (CO7500 Colourwave, Funakoshi Co., Ltd., Tokyo, Japan) at 590 nm. Subsequently, Lys-Au NCs/RB (0 (absence) and 1 μg/mL) was added to the *S. mutans* suspension and dispensed into 48-well microplates. After illumination by the white LED light for 1 min (as described in subsection 2.7) followed by incubation for 24 h, *S. mutans* suspensions were diluted 10-fold in a fresh medium and spread onto a blood agar medium (KYOKUTO Pharmaceutical Industrial, Co., Ltd., Sapporo, Japan). CFUs of *S. mutans* were examined after anaerobic incubation of the agar plate at 37 °C for 24 h.

To assess the dose-dependent antibacterial effects, the Lys-Au NCs/RB conjugate (0 (absence), 0.01, 0.1, and 1 μg/mL) was added to the suspension of *S. mutans* and dispensed into 48-well microplates. This suspension was illuminated by white LED light for 0 (no irradiation) or 1 min (as described in subsection 2.7) before incubation. After 24 h of incubation, the turbidity of each suspension was obtained using a colorimeter at 590 nm. To assess the effects of different light irradiation intervals, various light exposure times (0 (no irradiation), 30, 60, and 90 s) were applied to the *S. mutans* + Lys-Au NCs/RB (1 μg/mL) mixtures, and then the bacterial turbidity (following incubation for 24 h) was measured using a colorimeter at 590 nm.

To examine the aPDT effects of the Lys-Au NCs/RB conjugate on various bacterial species, Lys-Au NCs/RB (0 (absence) and 1 μg/mL) was added to suspensions of *E. coli*, *A. naeslundii*, *P. gingivalis*, and *P. intermedia*; the resulting mixtures were dispensed into 48-well microplates and then photoexcited for 1 min (as described in subsection 2.7). After anaerobic incubation for 24 h, the turbidity of each suspension was determined using a colorimeter at 590 nm.

### Morphological Observations

Lys-Au NCs/RB conjugate (0 (absence) and 1 μg/mL) was added to a suspension of *S. mutans* and photoexcited with a white LED for 1 min (as described in subsection 2.7)*.* After incubation for 24 h, samples were fixed using 2.5% glutaraldehyde in 0.1 M sodium cacodylate buffer (pH 7.4). After dehydration by passage through a series of solutions with increasing ethanol concentrations, critical point drying, and Pt–Pd coating, the morphology of the samples was observed using a scanning electron microscope (SEM; Model S-4000; Hitachi, Ltd., Tokyo, Japan) at an accelerating voltage of 10 kV. In addition, fixed samples were postfixed in 1% OsO_4_ and 0.1 M sodium cacodylate buffer (pH 7.4) at 4 °C for 1 h and then treated with ethanol and propylene oxide. After embedding the samples, they were sectioned and analyzed using a transmission electron microscope (TEM; Model JEM-1400; JEOL Ltd., Tokyo, Japan) at an accelerating voltage of 80 kV.

### Effect of Lys-Au NCs/RB Conjugate on *S. mutans* Biofilms

A suspension of *S. mutans* was dispensed into 48-well microplates to produce biofilms. After incubation for 24 or 48 h (permitting the formation of biofilms on the well bottoms), the spent medium was replaced with a fresh medium with or without Lys-Au NCs/RB (0 (absence) and 1 μg/mL, respectively). As the bacterial cells adhered to the plate surface, the replacement medium did not displace their biofilms. The plate was then subjected to white LED light irradiation for 1 min (as described in subsection 2.7). Immediately following irradiation, the contents of each well were stained using a LIVE/DEAD BacLight Bacterial Viability Kit (Thermo Fisher Scientific, Waltham, MA, USA), according to the manufacturer’s instructions. This procedure stains live bacteria with SYTO 9, which emits green fluorescence, while bacteria with damaged membranes are stained with propidium iodide, which emits red fluorescence. The bottom surface of each well of the microplate was observed using a fluorescence microscope (Model BZ-9000 BioRevo; Keyence Corporation, Osaka, Japan). Fluorescence intensity was measured using ImageJ software (version 1.41; National Institutes of Health, Bethesda, MD, USA).

In addition, biofilm formation by *S. mutans* was assessed using a biofilm formation assay kit (Dojindo Laboratories, Mashiki, Japan). A suspension of *S. mutans* was dispensed into a 96-well microplate at 180 μL/well. The plate was then fitted with a 96-peg lid. After 24 h of incubation (permitting the formation of biofilm on the pegs), the fresh medium with or without Lys-Au NCs/RB (0 (absence) and 1 μg/mL, respectively) was dispensed into a separate 96-well plate, and the peg lid was transferred from the first 96-well plate to a second plate containing the fresh medium. The lid-plate combination was then subjected to white LED light irradiation for 1 min (as described in subsection 2.7) and then placed in the incubator for continued incubation. After anaerobic incubation for 48 h, the amount of biofilm on each peg was quantified using the crystal violet method.^50^ The absorbance at 590 nm was measured on a microplate reader (Multiskan FC; Thermo Fisher Scientific).

### Cytotoxicity Assessments

The Lys-Au NCs/RB conjugate was added (to final concentrations of 0 (absence), 0.01, 0.1, and 1 μg/mL) to a suspension containing 1 × 10^4^ fibroblastic NIH3T3 cells (RIKEN BioResource Center, Tsukuba, Japan) and a culture medium (MEM alpha, GlutaMAX-I; Thermo Fisher Scientific) supplemented with 10% fetal bovine serum (Qualified FBS; Thermo Fisher Scientific) and 1% antibiotics (penicillin–streptomycin; Thermo Fisher Scientific). The suspensions were dispensed into 96-well microplates at 200 μL/well, irradiated with a white LED for 1 min (as described in subsection 2.7), and incubated at 37 °C in a 5% CO_2_ environment. Non-irradiated suspensions were examined as a control. After incubation for 24 h, the cytotoxicity was determined using the water-soluble tetrazolium salt (WST)-8 assay (Cell Counting Kit-8, Dojindo Laboratories) and the lactate dehydrogenase (LDH) assay (Cytotoxicity LDH Assay Kit-WST, Dojindo Laboratories) according to the manufacturer’s instructions. The absorbance at 450 nm (WST-8) or 490 nm (LDH) was measured using a microplate reader.

In addition, immunofluorescence double staining of vinculin and actin was performed. The cultured fibroblastic cells were washed with phosphate-buffered saline (PBS) for 5 min with 3.5% formaldehyde in PBS and permeabilized with 0.5% Triton X-100 for 10 min. Cells were incubated for 30 min with 1% bovine serum albumin (BSA) (7.5% w/v, obtained as albumin Dulbecco’s-PBS(−) solution, from bovine serum; FUJIFILM Wako Pure Chemical Corporation) and washed with PBS. Next, samples were incubated with shaking for 1 h at 37 °C in a mixture of 4.0 μg/mL anti-vinculin monoclonal antibody (Anti-Vinculin Alexa Fluor 488; eBioscience, San Diego, CA, USA), 0.12 μg/mL phalloidin (Acti-stain 555 fluorescent phalloidin; Cytoskeleton, Inc., Denver, CO, USA) dissolved in methanol, and 6.0 μg/mL 4′,6-diamidino-2-phenylindole solution (Dojindo Laboratories) diluted in BSA. The sample was then incubated (without shaking) in the same mixture for 1 day at 4 °C. The cells were washed with PBS and covered with a coverslip for observation using fluorescence microscopy.

Immunofluorescence staining was then conducted using the LIVE/DEAD Viability/Cytotoxicity Kit for mammalian cells (Thermo Fisher Scientific) according to the manufacturer’s instructions. Live cells are stained with calcein acetoxymethyl, resulting in green fluorescence, while cells with damaged membranes are stained with ethidium homodimer-1, resulting in red fluorescence. Stained samples were observed using fluorescence microscopy.

### Statistical Analysis

Statistical analysis was performed using one-way ANOVA with Tukey’s HSD post hoc test or using an unpaired Student’s *t* test. *P* < 0.05 was considered statistically significant. Statistical procedures were performed using the SPSS software package (version 11.0; IBM Corporation, Armonk, NY, USA).

## Abbreviations

aPDT: antibacterial photodynamic therapy
au: arbitrary unit
BSA: bovine serum albumin
CFU: colony forming units
DMF: dimethylformamide
DLS: dynamic light scattering
LDH: lactate dehydrogenase
LED: light emitting diode
Lys: lysozyme
Lys-Au-NC: lysozyme-gold nanoclusters
MTX: methotrexate
MB: methylene blue
NIR: near infrared
PBS: phosphate buffered saline
PL: photoluminescence
ROS: reactive oxygen species
RET: resonance energy transfer
RB: rose bengal
SEM: scanning electron microscope
TEM: transmission electron microscope
UV–vis: ultraviolet–visible
WST: water-soluble tetrazolium
